# Ab initio and template-based prediction of multi-class distance maps by two-dimensional recursive neural networks

**DOI:** 10.1186/1472-6807-9-5

**Published:** 2009-01-30

**Authors:** Ian Walsh, Davide Baù, Alberto JM Martin, Catherine Mooney, Alessandro Vullo, Gianluca Pollastri

**Affiliations:** 1 School of Computer Science and Informatics, University College Dublin, Dublin, Ireland; 2 Complex and Adaptive Systems Laboratory, University College Dublin, Dublin, Ireland

## Abstract

**Background:**

Prediction of protein structures from their sequences is still one of the open grand challenges of computational biology. Some approaches to protein structure prediction, especially *ab initio *ones, rely to some extent on the prediction of residue contact maps. Residue contact map predictions have been assessed at the CASP competition for several years now. Although it has been shown that exact contact maps generally yield correct three-dimensional structures, this is true only at a relatively low resolution (3–4 Å from the native structure). Another known weakness of contact maps is that they are generally predicted ab initio, that is not exploiting information about potential homologues of known structure.

**Results:**

We introduce a new class of distance restraints for protein structures: multi-class distance maps. We show that C_*α *_trace reconstructions based on 4-class native maps are significantly better than those from residue contact maps. We then build two predictors of 4-class maps based on recursive neural networks: one ab initio, or relying on the sequence and on evolutionary information; one template-based, or in which homology information to known structures is provided as a further input. We show that virtually any level of sequence similarity to structural templates (down to less than 10%) yields more accurate 4-class maps than the ab initio predictor. We show that template-based predictions by recursive neural networks are consistently better than the best template and than a number of combinations of the best available templates. We also extract binary residue contact maps at an 8 Å threshold (as per CASP assessment) from the 4-class predictors and show that the template-based version is also more accurate than the best template and consistently better than the ab initio one, down to very low levels of sequence identity to structural templates. Furthermore, we test both ab-initio and template-based 8 Å predictions on the CASP7 targets using a pre-CASP7 PDB, and find that both predictors are state-of-the-art, with the template-based one far outperforming the best CASP7 systems if templates with sequence identity to the query of 10% or better are available. Although this is not the main focus of this paper we also report on reconstructions of C_*α *_traces based on both ab initio and template-based 4-class map predictions, showing that the latter are generally more accurate even when homology is dubious.

**Conclusion:**

Accurate predictions of multi-class maps may provide valuable constraints for improved *ab initio *and template-based prediction of protein structures, naturally incorporate multiple templates, and yield state-of-the-art binary maps. Predictions of protein structures and 8 Å contact maps based on the multi-class distance map predictors described in this paper are freely available to academic users at the url .

## Background

Although a protein can be first characterised by its amino acid sequence, most proteins fold into three-dimensional structures that encode their function. Genomics projects leave us with millions of protein sequences, currently ≈ 6 × 10^6^, of which only a small fraction (≈ 1%) have their 3D structure experimentally determined. In the future, we might devolve upon structural genomics projects [1] the task to bridge the huge gap between sequence and structure. So far, though, even high throughput pipelines have not been able to make a dent in the massive ratio (roughly 120:1 and still growing) between known sequences and structures, and a large fraction of structures are found to be unsuitable for structural determination with the methods available [2]. Therefore, computational protein structure prediction remains an irreplaceable instrument for the exploration of sequence-structure-function relationships. Moreover, even if the structural genomics goal of providing a model for each fold is achieved, algorithms that are able to model protein structures based on putative homologues (the so called template-based methods) will become even more important to fully harness this novel knowledge. This is especially important for analyses at genomic or inter-genomic level, where informative structural models need to be generated for thousands of gene products in reasonable amounts of time.

Template-based algorithms for protein structure prediction typically adopt heuristics based on sequence and/or structural similarity to model the unknown structure of a protein (which we will refer to as query, or target) based on known structures that are fathomed to be homologous to it. Automating the modelling process is not trivial: there are several stages and critical points in the design (choice of templates, the creation of a correct structural alignement etc.) and for some of them manual intervention is at least helpful [3]. The use of multiple templates is also an open question (examples of methods using them are, for instance [4-6]).

The accuracy of template-based techniques strongly depends on the amount of detectable similarity between query and templates, thus preventing the reliable application of these methods to a significant fraction of unannotated proteins. This is the realm of the so called *ab initio *or de novo protein structure prediction (sometimes also termed "template-free modelling" [7]), where models are predicted not relying on similarity to proteins of known structure. Ab initio techniques are not nearly as accurate as those based on templates [3], but the design in this case is generally somewhat simpler. A system for the prediction of protein structures ab initio is usually composed of two elements: an algorithm to search the space of possible protein configurations to minimise some cost function; the cost function itself, composed of various restraints being either derived from physical laws, from structural features (e.g. secondary structure, solvent accessibility, residue contact maps) predicted by machine learning or other kinds of statistical system [8], or possibly constraints obtained from experiments. Fragment-based algorithms [9], that use fragments of proteins of known structure to reconstruct the complete structure of the target protein (i.e. to search the conformational space), have shown potential to improve ab initio predictions by confining the search to locally native-like structures.

Residue contact maps have been proposed as an intermediate representation between the primary sequence and the 3D structure of a protein and predicted by a score of different techniques [10-20]. Beyond the prediction of protein structure (e.g. [21]), contact maps have also been adopted to predict protein folding rates [22], protein disorder [23], and structure/function [24].

Most research has focussed on binary contact maps (i.e. two classes, contact or not). It is generally believed that binary maps provide sufficient information to unambiguosly reconstruct native or near-native models [10]. Nevertheless, the prediction quality of residue contact map predictors has not improved to satisfactory levels, despite years of attempts [25]. The main reason for this is perhaps that, if it is true that contact maps are roughly equivalent to protein structures, contact map prediction is, ultimately, roughly equivalent to protein structure prediction itself, with all its daunting complexity. More in detail: it is hard to learn long-range dependencies on contact maps, hence it is especially difficult to predict contacts between residues that have large sequence separations [3]; contact map prediction is an unbalanced problem, with far fewer contacts than non-contacts, especially for long-range contacts, for which the ratio between negative and positive examples can exceed 100; moreover contact map predictors are often ab initio, i.e. do not exploit information about homologues of known structure (a notable, recent exception is [26]). A more general problem with residue contact maps is that, although it has long been stated that native maps yield correct structures, this is true only at a relatively low resolution (3–4 Å on average, in the best case [10,27]).

In this article, we introduce a representation of protein structures based on a generalisation of binary contact maps, multi-class distance maps, and show that it is powerful and predictable with some success. Our tests suggest that exact 4-class maps (i.e. extracted from experimental structures), can quickly guide a simple optimisation search to nearly perfect models – significantly better than binary contact maps. We compare reconstructions based on binary and 4-class maps on a non-redundant set of 258 proteins of length between 51 and 200 residues. The reconstructions based on 4-class maps have an average Root Mean Square Deviation (RMSD) of roughly 2 Å and a TM-score of 0.83 to the native structure (4 Å and 0.65 for binary maps at 12 Å, which is the best-performing threshold in our tests).

We develop high-throughput systems for the prediction of 4-class distance maps, which exploit similarity to proteins of known structure, where available, in the form of simple structural frequency profiles from sets of PDB templates. These predictors are designed so that binary contact maps (at the CASP-mandated 8 Å threshold) can be trivially derived from them. We train two such predictors, by two-dimensional recursive neural networks: one ab initio, or relying on the sequence and on evolutionary information but no homology information from known structures; one in which homology information is provided as a further input.

We show that even very low sequence similarity to PDB templates (PSI-BLAST e-value up to 10) yields more accurate maps than the ab initio predictor. Furthermore, the predicted map is generally more accurate than the maps of the templates, suggesting that the combination of sequence and template information is more informative than templates alone.

Although this is not the main focus of this work, we also benchmark both ab initio and template-based multi-class map predictions for protein C_*α *_trace reconstruction, using a simple algorithm that relies only on the maps and on basic geometrical rules. We show that template-based traces are generally more accurate than ab initio ones even when homology is dubious, and that fair to accurate protein structure predictions can be generated for a broad range of homology to structures in the PDB.

## Results and discussion

In the first subsection of the results we briefly describe the quality of reconstructions of C_*α *_traces from native maps, and highlight that multi-class maps give rise to more accurate structures than binary contact maps. In the following subsection we describe new ab initio and template-based predictive systems for 4-class distance maps, and discuss their performances, and the performances of contact map predictors derived from them. In a third section we evaluate the contact map predictors thus developed against the methods that took part to the CASP7 competition. Finally, we briefly gauge the quality of C_*α *_traces reconstructed from predicted maps.

### Reconstruction of C_*α *_traces from native maps

The C_*α *_trace reconstruction protocol, which we describe in some detail in the Methods, is similar to that in [10] and essentially the same as in [27]. We should stress out that in the reconstruction protocol we only enforce the constraints encoded in the maps, plus very general geometrical rules (e.g. fixed distances between neighbouring C_*α *_atoms). If more constraints, for instance about short-range interactions, were taken into account, more accurate structural predictions would likely follow, and our results are to be considered a baseline for the resolution that maps can yield.

In the first set of simulations we compare the quality of reconstructions based on binary maps and multi-class maps for the case in which experimental constraints are known, that is the maps are native. We use binary maps at 12 Å, since these lead to more accurate results than a number of alternative we tested (tests not shown – similar conclusions in [28]).

In order to assess the quality of predictions, two measures are considered here: root mean square deviation (RMSD) and TM-score [29] between the predicted structure and the native one. TM-score assesses the similarity among two protein topologies based on their *C*_*α *_trace, is always in the [0, 1] interval (1 meaning a perfect structural match) and is independent on protein size. Two unrelated proteins have on average a TM-score of 0.17, while TM-scores above 0.4 are generally associated with structurally similar proteins [29]. For each protein in the test set, we run 10 folding simulations and select the best one. The results for the best simulations are then averaged over all the 258 proteins in the set and are reported in Table 1. While 12 Å maps produce structures at an average RMSD of 4 Å to the native, for multi-class maps this decreases to just over 2 Å. Running more than 10 reconstructions decreases both deviations, with those based on 12 Å maps plateauing around 3 Å and multi-class ones at a near perfect 1 Å.

**Table 1:** Reconstruction of C_*α *_traces from native maps

**Maps**	**RMSD**	**TM-score**
**Binary**	4.01	0.65
**4-Class**	2.23	0.83

Reconstruction algorithm results for the best models derived from binary and multi-class true contact maps.

### Multi-Class Distance Map Prediction

Only a small number of algorithms have been developed for the prediction of distance maps or parts thereof (e.g. [30,31]). Far more common are methods for the prediction of binary contact maps [11,13,14,16,18-20], with distance cutoffs of 6 Å, 8 Å, 10 Å, or 12 Å among the most common chosen to define the threshold between a contact and a non-contact. At the Critical Assessment of Protein Structure Prediction, CASP [7], maps are evaluated with a distance threshold of 8 Å between C_*β *_atoms (C_*α *_in the case of Gly), probably because a distance threshold of 8 Å is a good first approximation of physical contact. Nevertheless, there is some evidence that larger thresholds induce an easier reconstruction problem (e.g. [28,32]). There is a wide range of machine learning techniques for predicting contact maps: hidden markov models [15], recursive neural networks [17], multi-layer perceptrons [11,13,19], support vector machines [16,20], and self-organizing maps [18] are just a few. Predictors of contact maps are nearly always ab initio, meaning that they do not directly rely on similarity to proteins of known structure. In fact, often, much care is taken to try to exclude any detectable similarity between training and test set instances.

The method we present here is based on recursive neural networks, in particular 2-dimensional recursive neural networks (2D-RNNs). We predict both binary and 4-class maps. In the Methods section we give a detailed overview of the algorithms and experimental protocol.

The main objective of the experiments is to compare ab initio systems (PDB templates are assumed unavailable) and template-based systems. When very reliable PDB information (e.g. sequence identity to the query greater than 30–35%) is available we expect template-based predictions to be substantially better, and in fact, to nearly exactly replicate the maps of the best templates. More interesting questions are: whether template-based predictions improve on ab initio ones in the so called twilight zone of sequence similarity (20–30%) and in the midnight zone (less than 20%); whether, in these same regions, template-based predictions are better than can be obtained by simply copying the map of the best template, or a combination of the maps of the templates.

The base systems we test are multi-class ab intio (*M*_*AI*_) and multi-class with templates (*M*_*TE*_). From these systems we also derive (see Methods) binary map predictions at 8 Å for comparison with other predictors. We label these as 8_*AI *_and 8_*TE*_, for ab initio and template-based predictions, respectively.

Table 2 reports the comparison between the Q_2 _of 8 Å ab initio and template based predictions (8_*AI *_vs. 8_*TE*_) as a function of sequence identity to the best PDB hit. 8_*TE *_improves on 8_*AI *_for every level of sequence identity to the best template, except for the (0,10)% identity range in which the performances of the two systems are identical (Q_2 _= 98.1%). The gain is small in absolute value (0.2%) in the (10,20)% identity range, but this corresponds to a roughly 10% reduction of the number of errors. 8_*TE *_gains grow to 0.7% in the (10,20)% identity region (a 32% reduction in the number of errors), with performances stabilising at near perfect levels for higher sequence similarity (50–75% cut in errors).

**Table 2:** 8_*AI *_vs. 8_*TE*_

	10	20	30	40	50	60	70	80	90	≥ 90	All
8_*AI*_	98.1	97.5	97.8	97.8	98.0	97.7	98.1	97.7	98.0	98.2	97.9
8_*TE*_	98.1	97.7	98.5	98.9	99.2	99.3	99.5	99.3	99.5	99.7	98.9

Percentage of correctly classified residue pairs for the ab initio (8_*AI*_) and template based 8 Å predictor (8_*TE*_) as a function of sequence identity to the best template. Template sequence identity 10 means all proteins that have a best hit template in the identity range [0, 10) %, All is the complete set.

**Figure 1:**
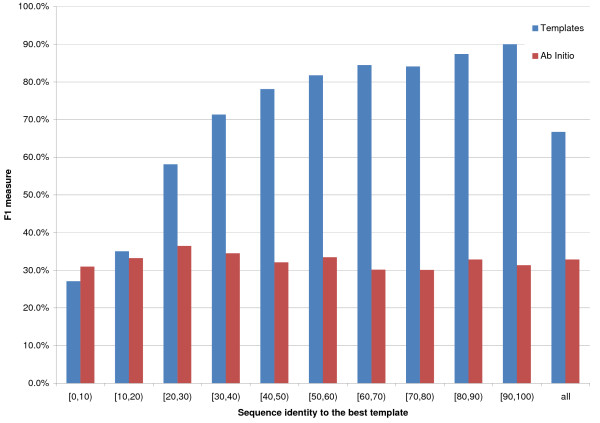
**8 Å prediction for sequence separation between 6 and 11**. On the x axis the sequence identity between the query and the best template. The bins' height is proportional to the average F1 for the contact class. Red bins represent ab initio predictions, while blue ones are template-based. Results for sequence separations between 6 and 11 residues, inclusive.

**Figure 2:**
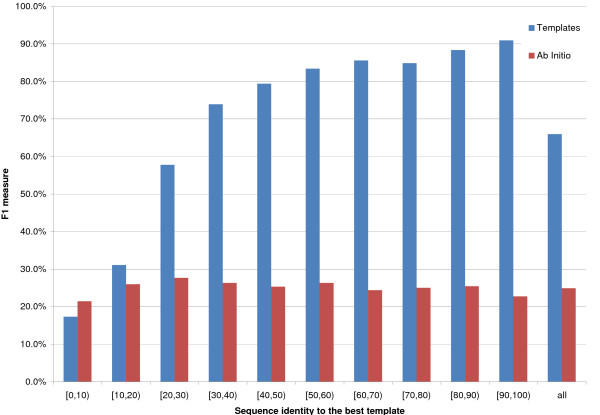
**8 Å prediction for sequence separation between 12 and 23**. On the x axis the sequence identity between the query and the best template. The bins' height is proportional to the average F1 for the contact class. Red bins represent ab initio predictions, while blue ones are template-based. Results for sequence separations between 12 and 23 residues, inclusive.

**Figure 3:**
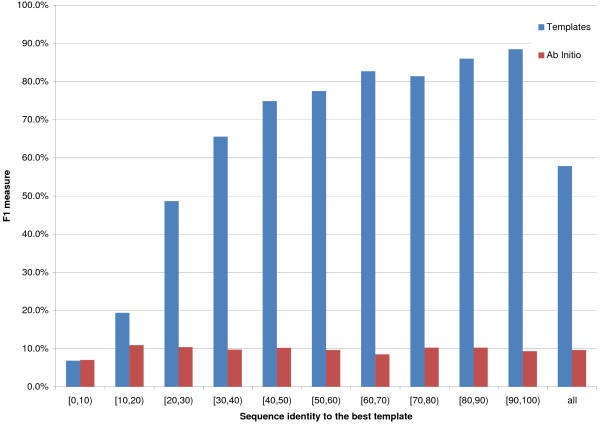
**8 Å prediction for sequence separation of 24 and greater**. On the x axis the sequence identity between the query and the best template. The bins' height is proportional to the average F1 for the contact class. Red bins represent ab initio predictions, while blue ones are template-based. Results for sequence separations of 24 and greater.

If one focusses only on the contact class, and in particular on contacts for sequence separations of [6, 11], [12, 23] and [24, ∞) residues (Figures 1, 2 and 3 report F1, or harmonic mean of Accuracy and Coverage, as a function of template identity for the three sequence separations), 8_*AI *_performs slightly better than 8_*TE *_if the best template shows a [0,10)% identity to the query, for sequence separations of [6, 11] and [12, 23] residues, but almost identically to 8_*TE *_for the largest sequence separation class. It is important to point out that approximately half of all proteins in this identity range have in fact no templates at all. For all other template identity ranges and sequence separations 8_*TE *_outperforms 8_*AI*_. For template identities of [20,30)% 8_*TE*_'s F1 is roughly 50% compared to just over 10% for the ab initio predictor.

Similarly, for multi class maps there is never a decrease in performance between *M*_*TE *_and *M*_*AI *_(Table 3 reports Q_4_), in fact there is a small gain of *M*_*TE *_over *M*_*AI *_even when the best template has a (0,10)% identity to the query.

**Table 3:** *M*_*AI *_vs. *M*_*TE*_

	10	20	30	40	50	60	70	80	90	≥ 90	All
*M*_*AI*_	72.1	66.2	69.8	70.1	70.4	67.9	72.9	66.6	69.9	71.1	70.0
*M*_*TE*_	72.6	69.1	83.0	89.1	92.1	92.6	95.1	93.5	95.5	96.7	87.2

Percentage of correctly classified residue pairs for the ab initio (*M*_*AI*_) and template based Multi class predictor (*M*_*TE*_) as a function of sequence identity to the best template. Template sequence identity 10 means all proteins that have a best hit template in the identity range [0, 10) %, All is the complete set.

**Figure 4:**
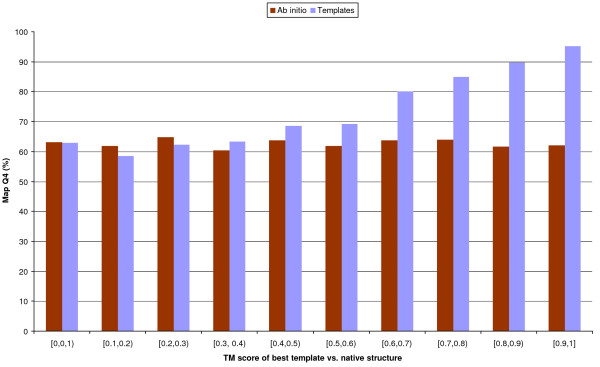
**4-class predictions vs. TM-score of the best template**. On the x axis is the TM-score between the query and the best template found by PSI-BLAST. The bins' height is proportional to the average *Q*_4 _of the map. Red bins represent ab initio predictions, while blue ones are template-based. Results for all sequence separations. The errors of individual bins are in the 0.7–2% range, with all differences greater than the sum of standard deviations of the template and ab initio bins except for the [0,0.1) interval.

In Figure 4 we report *M*_*TE *_and *M*_*AI *_Q_4 _as a function of the TM-score of the best template against the query's native structure. A TM-score of 0.4 or greater is deemed to indicate a clear structural relationship [29]. *M*_*TE *_outperforms *M*_*AI*_, on average, for TM-scores of 0.3 and above, while *M*_*AI *_performs better if the best template is in the 0.1–0.3 region. The two methods are tied again in the 0–0.1 region, but in most of these cases PSI-BLAST cannot find any template and both methods are effectively ab initio. 8_*TE *_and 8_*AI *_show the same trends as the 4-class predictors.

Prediction Q_2 _and Q_4 _for regions not covered by the templates are reported in Tables 4 and 5. In these areas the 8 Å predictions are virtually identical for the ab initio and template-based case, while for multi-class predictions there is a small difference, with an average improvement of 0.3% for *M*_*TE *_over *M*_*AI*_. This may be due to the easier contextual propagation in the multi-class case (the narrower class ranges impose stricter distance constraints among neighbours).

**Table 4:** 8_*AI *_vs. 8_*TE *_on non-template regions

	10	20	30	40	50	60	70	80	90	≥ 90	All
8_*AI*_	98.2	97.9	98.3	98.7	98.9	99.0	99.0	98.4	98.8	99.0	98.2
8_*TE*_	98.3	97.9	98.3	98.8	98.9	99.0	99.0	98.5	98.9	99.0	98.3

Identical to Table 2 except only calculated for non template regions of the map.

**Table 5:** *M*_*AI *_vs. *M*_*TE *_on non-template regions

	10	20	30	40	50	60	70	80	90	≥ 90	All
*M*_*AI*_	72.6	66.8	72.7	79.5	79.3	80.5	81.1	77.2	79.1	81.4	71.8
*M*_*TE*_	73.0	66.1	73.9	80.7	81.0	79.2	81.4	78.8	81.2	83.6	72.1

Identical to Table 3 except only calculated for non template regions of the map.

Tables 6 and 7 report the comparison of Q_2 _and Q_4 _between template based predictions and a baseline for 8 Å and multi-class respectively. The baseline simply calculates the distance class for position (*i*, *j*) from the coordinates in the best template. This means that the distance between the *i*-th and *j*-th residues is assumed to be the same as that between the corresponding (aligned) residues in the best template available (this being the one with the lowest PSI-BLAST e-value). We also tested different baselines in which, instead of just the top template, the top 10 templates and all templates were used to get the class by a majority vote among the templates covering each template. We tested both an unweighed vote and a vote in which each template is weighed by its sequence similarity to the query, cubed. The latter weighing scheme is identical to the one used to present the templates to the neural networks. In all cases the baseline is worse than the best hit baseline. We only report the predictions vs. baseline for the [0,30)% templates, since above 30% identity, as expected, the results are undistinguishable. In this twilight region, where it is non-trivial to extract information from templates, both 8_*TE *_and *M*_*TE *_outperform the baseline by clear margins. 8_*TE*_'s F1 on contacts is also higher than the baseline's by roughly 4%, 3% and 1% in the [0,10)%, [10,20)% and [20,30)% template identity ranges, respectively.

**Table 6:** 8_*TE *_vs. best template

	10	20	30
8_*TE*_	96.6	97.5	98.6
Baseline	94.6	96.4	98.3

Percentage of correctly classified residue pairs for 8_*TE *_when only considering the residues covered by the best template. Baseline is a predictor that copies the contact assignment from the best hit template. Template sequence identity 10 means all proteins that have a best hit template in the identity range [0, 10) %.

**Table 7:** *M*_*TE *_vs. best template

	10	20	30
*M*_*TE*_	69.3	74.5	86.0
Baseline	63.8	72.3	85.3

Percentage of correctly classified residue pairs for *M*_*TE *_when only considering residues covered by the best template. Baseline is a predictor that copies the class assignment from the best hit template. Template sequence identity 10 means all proteins that have a best hit template in the identity range [0, 10) %

In summary template-based systems outperform the baseline (Tables 6, 7), always improve on ab initio predictions (Tables 2, 3), and even improve slightly, on average, in non-template regions (Tables 4, 5). This suggests that it is possible to combine information from the sequence and from templates to produce contact map predictions that are more accurate than those that can be produced from either source. The fact that improvements over ab initio occur down to essentially junk templates (5–10% sequence identity by PSI-BLAST), seems to suggest that information beyond genuine homology can still be harnessed. When we applied a similar technique for exploiting template information to predict secondary structure and relative solvent accessibility [33] we only observed gains for higher (greater than about 15%) sequence identities. One possible reason for this difference is that ab initio predictions of secondary structure and solvent accessibility are, on average, quite accurate, while ab initio predictions of contact maps are fairly poor, especially for the contact class.

Finally, we looked at prediction accuracy for different classes of proteins, specifically all-alpha vs. all-beta. While all-alpha proteins are predicted slightly more accurately ab initio, the difference becomes marginal (and favours all-beta) when templates are introduced.

**Figure 5:**
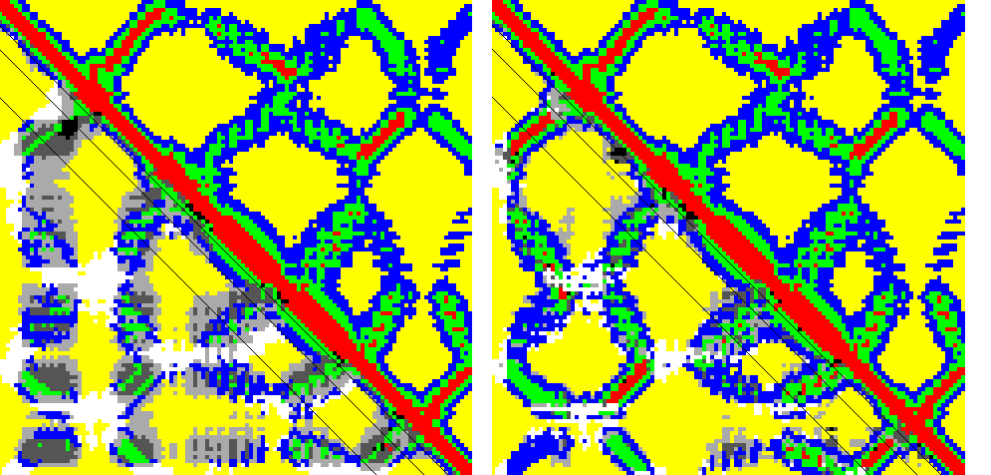
**An example of ab initio and template-based 4-class map prediction**. Protein 1B9LA Multi class contact maps for ab initio (left) and template-based (right) predictions. The best template sequence identity is 22.7%. The top right of each map is the true map and the bottom left is predicted. In the predicted half red, blue, green and yellow correspond to class 0, 1, 2 and 3 respectively. The greyscale in the predicted half corresponds to falsely predicted classes. The three black lines correspond to |*i *- *j*| = 6, 12, 24.

Figure 5 shows an example of a 4-class map predicted for a low best hit sequence identity of 22.7% over 120 residues. The top right of either map is the native map and the bottom left is predicted, ab initio for the map on the left side of the picture, based on templates for the map on the right. Red, blue, green and yellow correspond to class 0, 1, 2 and 3 respectively ([0,8) Å, [8,13) Å, [13,19) Å and [19,∞) Å). The greyscale in the predicted half corresponds to falsely predicted residue pairs. The three black lines correspond to |*i *- *j*| ≥ 6, 12, 24. While the ab initio prediction contains large greyscale areas (errors), the template-based prediction is nearly perfect.

### Contact Map prediction for CASP7 targets

We also tested 8_*AI *_and 8_*TE *_on the CASP7 targets [25], for comparison with the state of the art.

Comparisons at CASP7 were run with numerous restrictions, ultimately resulting in only 19 targets being considered, only contacts at a sequence separation of 24 or more residues being assessed (at least to identify the top predictors) and within these, only the *L*/5 top ranked contacts (where *L *is the protein length) being considered. Although we do not mean to question the merit of the ranking (i.e., the predictors that scored best are in fact likely to be the best available), we here substantially relax its criteria for two reasons: to be able to assess our predictors also on targets for which templates were available, given that 8_*TE *_is precisely designed for these; to focus on all sequence separations (excluding the trivial contacts within residues less than 6 positions apart), and on whole maps rather than a very small number of contacts. We measure performances by F1 (harmonic mean of Accuracy and Coverage – see Methods for details) on the contact class. We report the results for sequence separations of 6 to 11 residues, 12 to 23 residues, and 24 residues or greater, in tables 8, 9 and 10 respectively. In all of them we report results for all the 93 targets, but also results split by sequence identity (in 10% bins) to the best PSI-BLAST template in the PDB as available on April 22 2006, just before the beginning of the CASP7 experiment.

**Table 8:** CASP7 results, [6, 11] residue separation

template ID	[0,10)%	[10,20)%	[20,30)%	[30,40)%	[40,50)%	[50,60)%	[60,70)%	[70,80)%	All
proteins	29	29	16	4	7	4	3	1	93

positive pairs	3318	1990	1696	772	636	744	254	158	9568

negative pairs	60204	49856	43628	13682	13416	13146	3838	3112	200882

SAM_T06	12.2%	8.4%	9.4%	18.6%	12.5%	12.4%	11.5%	9.4%	11.0%

Betapro	26.0%	20.2%	27.8%	**48.2%**	26.9%	23.2%	27.9%	19.4%	25.7%

*ProfCon*	*34.1%*	*31.4%*	*29.7%*	*40.2%*	*32.7%*	*32.3%*	*37.7%*	*41.2%*	*33.3%*

Possum	17.2%	15.0%	18.5%	22.2%	21.5%	13.6%	18.3%	24.0%	17.4%

SVMcon	25.6%	25.0%	20.1%	23.6%	22.7%	25.9%	32.0%	36.7%	24.7%

8_*AI*_	**37.0%**	**30.2%**	**31.7%**	47.6%	**45.8%**	**48.7%**	**39.6%**	**59.6%**	**37.2%**

8_*TE*_	34.5%	41.6%	54.2%	78.4%	77.7%	91.1%	95.8%	92.8%	53.3%

Results for the CASP7 targets: sequence separations of 6 to 11 residues, inclusive. Comparison between our two predictors (8_*AI *_and 8_*TE*_) and the predictors ranked highest at CASP7. We report F1 for the contact class, as a function of sequence similarity to the best PSI-BLAST template. Predictions are from the CASP7 web site. ProfCon is in italics because it predicted 73 out of 93 maps, hence its results are not exactly comparable. In bold is the highest F1 of all predictors, excluding ProfCon, and excluding 8_*TE*_, which uses templates and has the highest F1 in all ranges except [0,10)% where it is slightly worse than only 8_*AI*_.

**Table 9:** CASP7 results, [12, 23] residue separation

template ID	[0,10)%	[10,20)%	[20,30)%	[30,40)%	[40,50)%	[50,60)%	[60,70)%	[70,80)%	All
proteins	29	29	16	4	7	4	3	1	93

positive pairs	3676	2336	3006	818	800	804	268	176	11884

negative pairs	117104	95092	84186	27010	26008	25896	7484	6148	388928

SAM_T06	13.6%	11.7%	18.1%	21.5%	16.7%	15.1%	12.7%	12.2%	14.6%

Betapro	18.5%	18.3%	24.4%	34.3%	20.4%	17.6%	25.9%	10.6%	21.4%

*ProfCon*	*26.3%*	*26.3%*	*25.2%*	*29.9%*	*25.8%*	*26.0%*	*27.5%*	*28.3%*	*26.3%*

Possum	14.8%	18.3%	24.0%	19.6%	18.1%	5.7%	15.3%	18.2%	17.8%

SVMcon	22.1%	22.3%	20.8%	21.5%	23.1%	22.0%	20.0%	40.8%	22.2%

8_*AI*_	**25.9%**	**25.8%**	**25.7%**	**40.7%**	**34.8%**	**35.6%**	**31.0%**	**47.3%**	**28.5%**

8_*TE*_	24.0%	40.2%	64.9%	82.8%	76.9%	91.3%	91.8%	86.5%	52.6%

Results for the CASP7 targets: sequence separations of 12 to 23 residues, inclusive. Comparison between our two predictors (8_*AI *_and 8_*TE*_) and the predictors ranked highest at CASP7. We report F1 for the contact class, as a function of sequence similarity to the best PSI-BLAST template. Predictions are from the CASP7 web site. ProfCon is in italics because it predicted 73 out of 93 maps, hence its results are not exactly comparable. In bold is the highest F1 of all predictors, excluding ProfCon, and excluding 8_*TE*_, which uses templates and has the highest F1 in all ranges except [0,10)% where it is slightly worse than only 8_*AI*_.

**Table 10:** CASP7 results, 24 or more residue separation

template ID	[0,10)%	[10,20)%	[20,30)%	[30,40)%	[40,50)%	[50,60)%	[60,70)%	[70,80)%	All
proteins	29	29	16	4	7	4	3	1	93

positive pairs	13532	8254	9720	2882	2972	2898	1046	644	41948

negative pairs	1163932	685474	887304	300968	244838	353646	47386	65662	3749210

SAM_T06	9.6%	9.7%	**13.9%**	10.5%	15.3%	8.5%	**12.6%**	3.6%	11.0%

Betapro	6.8%	7.9%	11.4%	10.8%	9.5%	6.0%	7.9%	2.9%	8.6%

*ProfCon*	*11.1%*	*13.3%*	*17.2%*	*15.9%*	*13.9%*	*9.7%*	*10.4%*	*8.0%*	*13.2%*

Possum	8.2%	9.0%	11.7%	8.7%	9.8%	3.4%	6.5%	0.5%	8.9%

SVMcon	**10.4%**	**13.2%**	12.9%	8.9%	14.4%	11.1%	11.3%	5.1%	**11.8%**

8_*AI*_	9.1%	10.4%	11.6%	**13.3%**	**18.4%**	**15.6%**	9.0%	**10.4%**	11.2%

8_*TE*_	8.6%	29.0%	53.1%	72.0%	80.3%	78.9%	92.5%	75.8%	37.6%

Results for the CASP7 targets: sequence separations 24 residues or more, inclusive. Comparison between our two predictors (8_*AI *_and 8_*TE*_) and the predictors ranked highest at CASP7. We report F1 for the contact class, as a function of sequence similarity to the best PSI-BLAST template. Predictions are from the CASP7 web site. ProfCon is in italics because it predicted 73 out of 93 maps, hence its results are not exactly comparable. In bold is the highest F1 of all predictors, excluding ProfCon, and excluding 8_*TE*_, which uses templates and has the highest F1 in all ranges except [0,10)% where it performs worse than some of the predictors.

The first conclusion we can derive from the tables is that our ab initio predictor (8_*AI*_) is state-of-the-art.

In the 6 to 11 and 12 to 23 residue separation regions it outperforms all other predictors for which a direct comparison is possible both on all 93 targets and on the 29 targets for which PSI-BLAST only finds PDB hits at 10% sequence identity or less. For template identity of [0,10)% in the [12, 23] sequence separation table ProfCon [19] has a slightly higher F1 (26.3% vs. 25.9%) but the results are not directly comparable because ProfCon did not predict 4 of the 29 proteins in this bin. In the [24, ∞) separation region 8_*AI *_performs best in 4 of the 8 bins, and only slightly worse than SVMcon [20] (11.8% vs. 11.2%) on the 93 targets, and slightly worse than SVMcon (10.4% vs 9.1%) and than SAM_T06 [34] (9.6% vs. 9.1%) on the 29 targets with [0,10)% identity PSI-BLAST templates.

8_*TE*_'s performances on the 93 CASP7 targets essentially confirm what we found on the larger S3129 set: there is a small decrease in F1 for the [0,10)% template identity class with respect to 8_*AI *_(37% to 34.5%, 25.9% to 24% and 9.1% to 8.6% for sequence separations of [6, 11], [12, 23] and [24, ∞), respectively); overall the predictions are far more accurate than those of the other predictors, none of which exploits templates; in the the [10,20)% template identity class there is already a very large gain compared to the second best (41.6% vs. 30.2% for [6, 11] separation, 40.2% vs. 25.8% for [12, 23] and 29% vs. 13.2% for [24, ∞) – 8_*AI *_being the second best in the first two classes, and SVMcon in the third one).

Because of the way our predictor is designed Accuracy ≈ Coverage ≈ F1 (more details in Methods). For the other algorithms the trade-off between Coverage and Accuracy varies with some predicting more contacts (SAM_T06 and to an extent SVMcon) thus showing a higher Coverage and lower Accuracy, and the others being more balanced. Overall only in a handful of cases does a method's Coverage exceed its Accuracy by more than 2 to 1: SAM_T06 for [12, 23] separation (Accuracy = 8.9%, Coverage = 40.2%); SAM_T06 and SVMcon for 24+ separation (Accuracy = 6.9%, Coverage = 27% and Accuracy = 8.5%, Coverage = 19%, respectively). The opposite happens only once: Possum for 24+ separation (Accuracy = 16%, Coverage = 6.2%).

### Modelling protein structures from predicted maps

**Figure 6:**
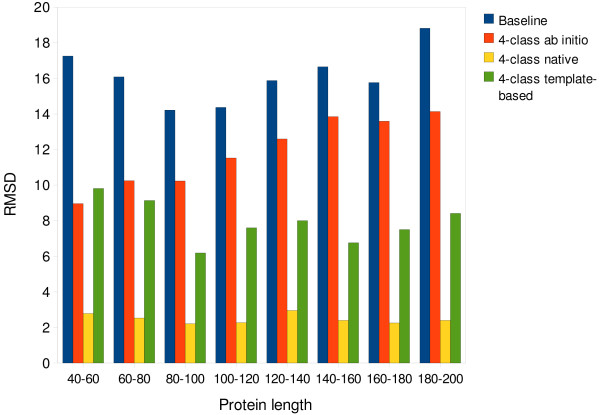
**Reconstructions from 4-class contact maps**. Average RMSD vs. sequence length is shown for models derived from true 4-class maps (yellow bins), from 4-class maps predicted using information derived from homologues (*M*_*TE*_) (green bins) and from 4-class maps predicted *ab initio *(red bins), together with the baseline (blue bins). Note that, since no templates are allowed that show a sequence identity greater than 95% to the query, the *M*_*TE *_results are based on a mixture of good, bad and no templates (see Figure 6 for a sample distribution of template quality). Standard deviations are approximately 1.3 Å for the 40–60 class, 1.1 Å for the 60–80 one and less than 1 Å for the other classes.

**Figure 7:**
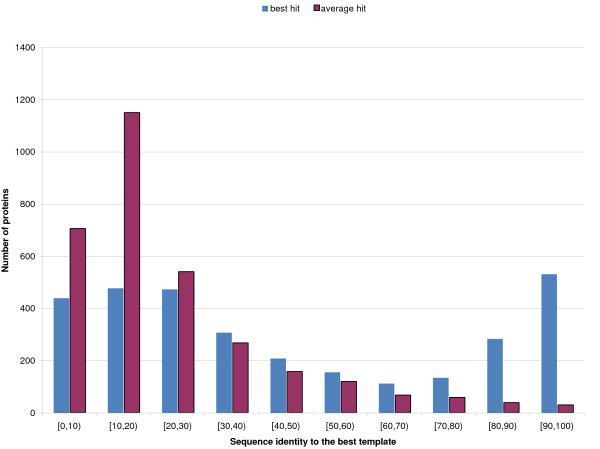
**Best and Average template distribution**. Distribution of best-hit (blue) and average (red) sequence similarity in the PSI-BLAST templates for the S3129 set. Hits above 95% sequence similarity excluded.

In Figure 6, the average RMSD vs sequence length is shown for models for set S258 derived from true 4-class contact maps (yellow bins), from *M*_*TE *_maps (green) and from *M*_*AI *_maps (red), together with the baseline (blue). The baseline represents a structure collapsed into its center of mass. Note that no templates are allowed that show a sequence identity greater than 95% to the query in order to prevent a structure from being reconstructed from its own PDB file. Hence, the *M*_*TE *_results are based on a mixture of good, bad and no templates, akin to the distribution one would expect when presented with a protein sequence that is not in the PDB. The distribution of template identity for S258 (not reported) resembles closely the one for the training/testing set, reported in Figure 7.

**Figure 8:**
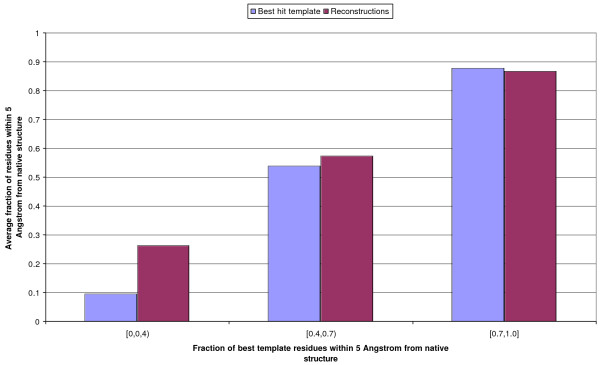
**Quality of 3D models from 4-class maps vs. TM-score of the best template**. On the x axis is the fraction of residues in the query which are within 5 Å of the template. The bins' height is proportional to the average fraction of residues in either the 3D model (red bins) or the best template (blue bins) that are within 5 Å of the native structure.

In Figure 8 we report the quality of reconstructions as a function of the TM-score between the best template and the query. We measure quality as the fraction of the native structure's residues that are modelled within 5 Å. When the template is perfect to near-perfect (TM-score above 0.7) the reconstruction from the map is, on average, very slighly worse (-1%) than the template. This is not surprising, as even from native maps model quality levels off at a TM-score of 0.83. When the TM-score between the best template and the native structure is between 0.4 and 0.7, models built from 4-class maps are slightly better than the templates (covering 4% more residues within 5 Å), and substantially better (+17%) when the best template has a TM-score under 0.4. If instead of residue coverage at 5 Å we measure the TM-score of the model and of the best template vs. the native structure (only focussing on the area covered by the best template) we obtain broadly similar results, but slightly less favourable for the models, with results now undistinguishable in the 0.4–0.7 region and still slightly worse in the 0.7–1 one. We can broadly conclude that, if good templates are available, reconstructions from 4-class maps are only about as good as them in the area covered by the template.

It is also important to note that the results are an average of 10 reconstructions. If more reconstructions were run and, especially, if these were ranked effectively, the results would improve considerably. The average reconstruction RMSD for *M*_*TE *_is 9.46 Å and the average TM-score 0.51 (Table 11). If the best of the 10 reconstructions is picked, these improve to 8.59 Å and 0.55, respectively.

Tested on both ab initio and template-based 4-class maps, our results show that template-based reconstructions are generally more accurate than ab initio ones even when homology is dubious. For sequence similarity above 30% the predictions' TM-score is on average slightly above 0.7, is 0.45 in the 20–30% interval, and 0.27 in the region below 20%. If reconstruction performances are measured on the S258 set without allowing homology information at any stage (pure ab initio predictions) the average TM-score is 0.27, with 43 of the 258 structures above a TM-score of 0.4.

**Table 11:** Reconstruction from ab initio and template-based 4-class maps

**Maps**	**RMSD**	**TM-score**
*M*_*AI*_	14.60	0.27
*M*_*TE*_	9.46	0.51

Reconstruction algorithm results for models derived from multi-class predicted contact maps with (*M*_*TE*_) and without (*M*_*AI*_) allowing homology information. Note that, since no templates are allowed that show a sequence identity greater than 95% to the query, the *M*_*TE *_results are based on a mixture of good, bad and no templates (see Figure 6 for a sample distribution of template quality). The reported values are the average over the 10 runs of simulated annealing.

## Conclusion

In this work we have described a machine learning pipeline for high-throughput prediction of protein contact maps, and have introduced a number of novel algorithmic ideas.

Based on the observation that protein binary contact maps are lossy representations of the structure and yield only relatively low-resolution models, we have introduced multi-class maps, and shown that, via a simple simulated annealing protocol and based on 10 reconstructions, these lead to much more accurate models than binary maps, with an average RMSD to the native structure of just over 2 Å and a TM-score of 0.83.

Extending on ideas we have developed for predictors of secondary structure and solvent accessibility [33] we have presented systems for the prediction of binary and 4-class maps that use structural templates from the PDB. 4-class maps lead to a more balanced prediction problem than binary ones. Although it is unclear whether because of this, or because of the nature of the constraints encoded into the maps, template-based systems for the prediction of 4-class maps we tested are capable of exploiting both sequence and structure information even in cases of dubious homology, significantly improving over their ab initio counterpart well into and below the twilight zone of sequence identity. 4-class map predictions are also far more accurate than the maps of the best templates for all the twilight and midnight zone of sequence identity. This also partly holds for 8 Å maps derived from 4-class predictors. Furthermore, we compared our 8 Å predictions with the best systems at the last CASP7, and showed that they are state-of-the-art, and, again, that even very low sequence similarity templates (10% or better) induce far better contact maps that all the ab initio predictors that performed best at CASP7.

Finally we have shown that template-based predictions of 4-class maps lead to fair predictions of protein structures, with an average TM-score of 0.7 or higher to the native when good templates are available (sequence identity greater than 30%), and of 0.45 in the [20%, 30%) identity region. Predictions for lower sequence identities to structural templates are still generally poor, at an average TM-score of 0.27. Nevertheless, it is important to note how: 1) the reconstruction protocol we use is basic, and would be likely improved by taking into account more than the interactions contained in the maps; 2) the component for homology detection in this study is basic (PSI-BLAST), and entirely modular, in that it may be substituted by any other method that finds templates without substantially altering the pipeline. Whether more subtle homology detection or fold recognition components could be substituted to PSI-BLAST, with or without retraining the underlying machine learning systems, is the focus of our current studies. The overall pipeline, including the template-based component, is available at the URL: . Protein structure predictions are based on 4-class maps and templates are automatically provided to the pipeline when available.

## Methods

### Optimisation Algorithm

The algorithm we use for the reconstruction of the coordinates of protein C_*α *_traces is essentially the one described in [27]. In this, in the first phase an extended, random (but clash-less) trace is generated, which is then refined with the aim of realising as many predicted contacts as possible. This is achieved by global optimisation of a potential (pseudo-energy) function using local moves and a simulated annealing protocol. The search strategy is similar to that in [10], but with two main differences: the form of the potential (see below); the fact that segments of the protein predicted to be in a helix are displaced as a whole, without modifying their geometry.

### Pseudo-energy function

Let $S_{n}$ = {*r*_*i*_}_*i *= 1...*n *_be a sequence of *n *3D coordinates, with *r*_*i *_= (*x*_*i*_, *y*_*i*_, *z*_*i*_) the coordinates of the *i*-th C_*α *_atom of a given conformation related to a protein *p*. Let $D_{S_{n}}$ = {*d*_*ij*_}_*i*<*j*_, *d*_*ij *_= ||*r*_*i *_- *r*_*j*_||_2_, be the corresponding set of *n*(*n - *1)/2 mutual distances between C_*α *_atoms. A first set of constraints *C *comes from the (predicted) contact map and depends on the type of contact maps, i.e. binary or multi class maps. The representation of protein models induces the constraints

ℬ = {*d*_*ij*_∈ [*D*_*B *_- 0.07, *D*_*B *_+ 0.07], |*i *- *j*| = 1} (*D*_*B *_= 3.803 Å), encoding bond lengths, and another set H = {*d*_*ij *_≥ *D*_*HC*_, *i *≠ *j*} for clashes (we set *D*_*HC*_, the minimum distance between *C*_*α *_atoms, to 4 Å). The set ℳ=C∪ℬ∪H defines the configurational space of physically realisable protein models.

When using binary contact maps the set of contraints coming from the predicted maps can be represented as a matrix $C=\{c_{ij}\}\in {\left\{0,1\right\}}^{n^{2}}$. Let $F_{0}$ = {(*i*, *j*) | *d*_*ij *_> *d*_*T *_∧ *c*_*ij *_= 1} denote the pairs of amino acid in contact according to *C *(binary case) but not in $S_{n}$ ("false negatives"). *d*_*T *_is the distance threshold between contacts and non-contacts, and is set to 12 Å in our tests. Similarly, define $F_{1}$ = {(*i*, *j*)| *d*_*ij *_≤ *d*_*T *_∧ *c*_*ij *_= 0} as the pairs of amino acids in contact in $S_{n}$ but not according to *C *("false positives"). The objective function is then defined as:

(1) $$\begin{array}{lll} C(S_{n},ℳ) & = & \alpha_{0}\{1+\sum_{(i,j)\in F_{0}}{\left(d_{ij}/d_{T}\right)}^{2}+\sum_{(i,j):d_{ij}\notin ℬ}{\left(d_{ij}-D_{B}\right)}^{2}\}\\ & + & \alpha_{1}|F_{1}|+\alpha_{2}\sum_{(i,j):d_{ij}\notin H}e^{\left(D_{HC}-d_{ij}\right)} \end{array}$$

In the case of 4-class contact maps, the constraint derived from the predicted map assumes a slightly different form. Since contacts between pairs of C_*α *_are here predicted in four classes, a contact is penalised not only if it is not present in the predicted map, but also depending on its distance to the boundaries of the corresponding class. Let $F_{k}$ = {(*i*, *j*)|*D*_*k *_<*d*_*ij *_<*D*_*k*+1_and *c*_*ij *_≠ *k*} with *D*_*k *_being the distance thresholds that define the classes. Let ${D^{\prime }}_{k}$ = (*D*_*k *_+ *D*_*k*+1_)/2, then the objective function is defined as:

(2) $$\begin{array}{lll} C(S_{n},ℳ) & = & \alpha_{0}\{1+\sum_{k}\sum_{(i,j)\in F_{k}}{\left(d_{ij}/{D^{\prime }}_{k}\right)}^{2}\\ & + & \sum_{(i,j):d_{ij}\notin ℬ}{\left(d_{ij}-D_{B}\right)}^{2}\}+\alpha_{1}\sum_{(i,j):d_{ij}\notin H}e^{\left(D_{HC}-d_{ij}\right)} \end{array}$$

In all the experiments, we run the annealing protocol using a linear schedule with initial (resp. final) temperature proportional to the protein size (resp. 0). Pseudo energy parameters are set to *α*_0 _= 0.2 (false non-contacts), *α*_1 _= 0.02 (false contacts) and *α*_2 _= 0.05 (clashes) for binary maps and *α*_0 _= 0.005 and *α*_1 _= 0.05 (clashes) for multi-class maps, so that the conformational search is biased towards the generation of compact clash-free structures and with as many of the predicted contacts realised.

### Recursive Neural Networks

2D-RNNs were previously described in [14] and [35]. This is a family of adaptive models for mapping two-dimensional matrices of variable size into matrices of the same size.

If *o*_*j*, *k *_is the entry in the *j*-th row and *k*-th column of the output matrix, and *i*_*j*, *k *_is the input in the same position, the input-output mapping is modelled as:

$$\begin{array}{lll} o_{j,k} & = & N^{\left(O\right)}\left(i_{j,k},h_{j,k}^{\left(1\right)},h_{j,k}^{\left(2\right)},h_{j,k}^{\left(3\right)},h_{j,k}^{\left(4\right)}\right)\\ h_{j,k}^{\left(1\right)} & = & N^{\left(1\right)}\left(i_{j,k},h_{j-1,k}^{\left(1\right)},..,h_{j-s,k}^{\left(1\right)},h_{j,k-1}^{\left(1\right)},..,h_{j,k-s}^{\left(1\right)}\right)\\ h_{j,k}^{\left(2\right)} & = & N^{\left(2\right)}\left(i_{j,k},h_{j+1,k}^{\left(2\right)},..,h_{j+s,k}^{\left(2\right)},h_{j,k-1}^{\left(2\right)},..,h_{j,k-s}^{\left(2\right)}\right)\\ h_{j,k}^{\left(3\right)} & = & N^{\left(3\right)}\left(i_{j,k},h_{j+1,k}^{\left(3\right)},..,h_{j+s,k}^{\left(1\right)},h_{j,k+1}^{\left(3\right)},..,h_{j,k+s}^{\left(3\right)}\right)\\ h_{j,k}^{\left(4\right)} & = & N^{\left(4\right)}\left(i_{j,k},h_{j-1,k}^{\left(4\right)},..,h_{j-s,k}^{\left(4\right)},h_{j,k+1}^{\left(4\right)},..,h_{j,k+s}^{\left(4\right)}\right)\\ & & j,k=1,...,N\\ & & s=1,...,S \end{array}$$

where $h_{j,k}^{\left(n\right)}$ for *n *= 1,..., 4 are planes of hidden vectors transmitting contextual information from each corner of the matrix to the opposite corner. We parametrise the output update, and the four lateral update functions (respectively N^(*O*) ^and N^(*n*) ^for *n *= 1,..., 4) using five two-layered feed-forward neural networks, as in [35]. Stationarity is assumed for all residue pairs (*j*, *k*), that is the same parameters are used across all *j *= 1,..., *N *and *k *= 1,..., *N*. Each of the 5 neural network contains its own individual parameters, that are not constrained to the ones of the other networks.

Since we are trying to predict both a 4-class map and a binary map, we model both classification problems within the same 2D-RNN. Hence the output *o*_*j*, *k *_will have two components:

$$o_{j,k}=(o_{j,k}^{\left(4\right)},o_{j,k}^{\left(2\right)})$$

where $o_{j,k}^{\left(4\right)}$ is a vector of four numbers representing the estimated probabilities of residues *j *and *k *belonging to each of the four distance classes, and $o_{j,k}^{\left(2\right)}$ is the same for the two binary (contact vs. non-contact) classes. Both components are implemented by (independent) softmax units.

As modelled in the input-output mapping equations above, we use 2D-RNNs with *shortcut connections*. This means that a memory state depends explicitly on more that the memory state immediately previous to it along the direction of contextual propagation, i.e. the memory span is greater than one. This is effective because gradient-based learning in deep layered architectures suffers from the well known vanishing gradient problem [36]. Allowing shortcuts of length *S *(i.e. the memory state in position *i *depends directly on the state in position *i *- *S*) creates new paths of roughly 1/*S *of the length of the ones induced by 1-step memory dependencies, thus facilitating the transmission of contextual information over larger distances. Indeed, shortcut connections can be placed starting at any of the previous states *i *- *s *for any *s *∈ 1,.., *S*. A selective placement of shortcuts was used to produce near perfect secondary structure predictions in a bidirectional recurrent neural network when (*i*, *s*) represent native contacts [37]. Notice that increasing the number of shortcuts increases the parameters resulting in a model that may more easily overfit the data. Extending the shortcut idea beyond the 2D case or in any direction of contextual propagation is straightforward. Shortcut directions and patterns are not strictly constrained (so long as cycles are not introduced in the directed graph representing the network) and may even be learned.

The choice of input *i*_*j*, *k *_is an important factor for the algorithm. In the case of contact map prediction the simplest input is the amino acid symbols at (*j*, *k*). Different input encodings can be constructed to improve the algorithm. For example, contact density was used in [38] to improve contact map prediction accuracy significantly. In the Input Design section we describe the input encoding we used in this study.

#### Training

Learning proceeds by gradient descent by minimising the relative cross entropy between target and output. Since there are two independent output components (a 4-class and a binary one), the error is in fact the sum of two cross entropies, which are weighed equally. Careful management of the gradient must take place, not letting it be too small or too large: the absolute value of each component of the gradient is kept within the [0.1,1] range, meaning that it is set to 0.1 if it is smaller than 0.1, and to 1 if it is greater than 1. The learning rate is set to 0.3 divided by the the total number of proteins in the dataset. The weights of the networks are initialised randomly.

Learning is slow due to the complexity of the problem. Each 2D-RNN contains 5 neural networks, replicated *N*^2 ^times for a protein of length *N*. During each training epoch forward and back-propagation has to occur in each of the 5 × *N*^2 ^networks, for all *P *proteins in the training set. The neural network forward and back-propagation have a complexity proportional to O(*θ*) where *θ *is the number of parameters in the network. Learning generally converges at about 300–350 epochs. Although the complexity of an epoch is polynomial at O(*θN*^2^*P*), the large size of the training set, and especially the quadratic term in the length of the proteins make learning quite time-consuming. Training of all systems (ab initio, template-based) took approximately three months on a cluster of 10 2.8 GHz CPUs.

However, during prediction only one forward propagation needs to run for each instance, meaning that predictions for a set may be run in roughly 3 orders of magnitude less time than a training on the same set. For instance, maps for 1000 proteins of average length 120 amino acids can be predicted in approximately 13 hours on a single 2.8 GHz CPU, and genomic-scale predictions are possible even on a small cluster of machines.

#### Architecture

In each of the 5 neural networks used to parameterise the functions, N^(*O*) ^and N^(*n*) ^for *n *= 1,..., 4, we use a single hidden layer. Let *N*_*hh *_and *N*_*ho *_denote the number of units associated with the hidden layer and the output layer of the hidden contextual networks respectively. From the definition of the 2D-RNN we see that each hidden network has *I *regular input units and 2 × *N*_*ho*_+ *S *× *N*_*ho *_contextual inputs, where *S *is the total number of shortcuts allowed. Thus, including the usual bias terms in each layer, the total number of parameters in one of the four hidden networks is: (*I *+ 2 ×*N*_*ho *_+ *S *× *N*_*ho*_) × *N*_*hh *_+ *N*_*hh *_+ *N*_*hh *_× *N*_*ho *_+ *N*_*ho*_. The output network also contains *I *regular inputs but it takes contextual inputs from the four hidden networks 4 × *N*_*ho *_resulting in: (*I *+ 4 × *N*_*ho*_) × *N*_*h *_+ *N*_*h *_+ *D *× *Nh*+ *D *parameters, where *N*_*h *_are the number of units in the hidden layer of the output network and *D *is the number of classes. Only the output units of the output network have softmax functions in order to estimate Bayesian posterior probability of class membership. All other units have tanh transfer functions.

No overfitting avoiding techniques such as early stopping or weight decay were applied given the very large size of the datasets, and the fact that we ensemble many networks in the final predictor (see below).

Due to the large computational power needed to train one model we ensemble networks both from different trainings and from different stages of the same training. Networks are saved every 5 epochs, and for each training the last 3 saved networks are ensembled. Three networks with different architectural parameters (*N*_*hh *_= *N*_*ho *_= *N*_*h *_= 13, 14, 15) are trained for each predictor. Results for network performances in this work are reported for these ensembles of 3 × 3 = 9 models. Ensembling leads to significant classification performance improvements over single models.

All results are in 5-fold cross validation, meaning that, in fact, 5 times 9 models are available for each system. For the reconstruction results (see next section) only the final networks for each training are ensembled, for a total of 1 × 3 × 5 = 15 for each system.

The number of classes is *D *= 4 + 2 (multi-class plus binary). For all networks the number of shortcuts is *S *= 2, with more sophisticated shortcut placements to be investigated in the future.

#### Input Design

Input *i*_*j*, *k *_associated with the *j*-th and *k*-th residue pair contains primary sequence information, evolutionary information, structural information, and direct contact information derived from the PDB templates:

(3) $$i_{j,k}=(i_{j,k}^{\left(E\right)},i_{j,k}^{\left(T\right)})$$

where, assuming that *e *units are devoted to evolutionary sequence information and structural information in the form of secondary structure [33,39], solvent accessibility [33,40] and contact density [38]:

(4) $$i_{i,j}^{\left(E\right)}=(i_{j,k}^{{\left(1\right)}^{\left(E\right)}},...,i_{j,k}^{{\left(e\right)}^{\left(E\right)}})$$

Template information is placed in the remaining *t *units:

(5) $$i_{j,k}^{\left(T\right)}=(i_{j,k}^{{\left(1\right)}^{\left(T\right)}},...,i_{j,k}^{{\left(t\right)}^{\left(T\right)}})$$

Hence *i*_*j*, *k *_contains a total of *e *+ *t *components.

In this work *e *= 58. 20 + 20 units correspond to the frequencies of residues observed in the two columns *j *and *k *of the multiple sequence alignment. Structural information in the form of secondary structure (three classes), solvent accessibility (two classes), and contact density (four classes) for residue *j *and *k *are placed in the remaining 6,4 and 8 input units respectively.

For the template units we use *t *= 5, representing weighted contact class information from the templates and one template quality unit. Assume that $d_{j,k}^{\left(p\right)}$ is a 4-component binary vector encoding the contact class of the (*j*, *k*)-th residue pair in the *p*-th template. Then, if *P *is the total number of templates for a protein:

(6) $$(i_{j,k}^{{\left(1\right)}^{\left(T\right)}},...,i_{j,k}^{{\left(4\right)}^{\left(T\right)}})=\frac{\sum_{p=1}^{P}w_{p}d_{j,k}^{\left(p\right)}}{\sum_{p=1}^{P}w_{p}}$$

where *w*_*p *_is the weight attributed to the *p*-th template. If the sequence identity between template *p *and the query is *id*_*p *_and the quality of a template (measured as X-ray resolution + R-factor/20 or 10 for NMR hits, as in [41]) is *q*_*s*_, then the weight is defined as:

(7) $$w_{p}=q_{p}id_{p}^{3}$$

Taking the cube of the identity between template and query allows us to drastically reduce the contribution of low-similarity templates when good templates are available. For instance a 90% identity template is weighed two orders of magnitude more than a 20% one. In preliminary tests (not shown) this measure performed better than a number of alternatives.

The final unit of *i*_*j*, *k*_, the quality unit, encodes the weighted average coverage and similarity of a column of the template profile as follows:

(8) $$i_{j,k}^{{\left(5\right)}^{\left(T\right)}}=\frac{\sum_{p=1}^{P}w_{p}c_{p}}{\sum_{p=1}^{P}w_{p}}$$

where *c*_*p *_is the coverage of the sequence by template *p *(i.e. the fraction of non-gaps in the alignment). Encoding template information for the binary maps is similar.

Ab initio based predictions use only the first part of the input, $i_{j,k}^{\left(E\right)}$ from equation 4, including secondary structure, solvent accessibility and contact density, although these are predicted ab initio. The template based predictions use the complete *i*_*j*, *k *_as input.

### Datasets

The data set used to train and test the predictors is extracted from the January 2007 25% pdb_select list [41]. We assign each residue's secondary structure and solvent accessibility using DSSP [42]. Exact secondary structure and solvent accessibility are used during training, but during testing we use predicted ones (see below). We remove all sequences for which DSSP does not produce an output, after which the set (S3129) contains 3129 proteins, 461,633 amino acids and just over 100 million residue pairs. Since training is computationally very demanding we create a reduced version of S3129 from which we exclude proteins longer than 200 residues. This set contains 2,452 proteins, and approximately 69 million residue pairs. Secondary structure is mapped from the eight DSSP classes into three classes as follows: H, G, I → Helix; E, B → Strand; S, T, → Coil. Relative solvent accessibility is mapped into four roughly equal classes: completely buried (0–4% exposed), partly buried (4–25% exposed), partly exposed (25–50%) and completely exposed (more than 50%). Contact Density [38] is defined as the principal eigenvector of a protein's residue contact map at 8 Å, multiplied by the principal eigenvalue, and is assigned to one of 4 roughly equal classes, corresponding to very low, medium-low, medium-high and very high density (see [38] for details). All systems are trained in 5-fold cross-validation. This is obtained by splitting S3129 into 5 approximately equal folds, then (for training purposes) removing from the folds all proteins longer than 200 residues. Testing is on the full folds, i.e. including proteins longer than 200 residues.

Evolutionary information in the form of multiple sequence alignments have long being shown to improve prediction of protein structural features [14,35,39,43-47]. Multiple sequence alignments for the proteins in the training/test set are extracted from the NR database as available on March 3 2004 containing over 1.4 million sequences. The database is first redundancy reduced at a 98% threshold, leading to a final 1.05 million sequences. The alignments are generated by three runs of PSI-BLAST [48] with parameters *b *= 3000, *e *= 10^-3 ^and *h *= 10^-10^.

We choose four distance classes as follows: [0,8) Å, [8,13) Å, [13,19) Å and [19, ∞) Å. The first class roughly corresponds to physical contacts, and matches the standard contact class adopted at CASP [25], the two middle classes were chosen to be roughly equally numerous and to span all categories of possible interaction (in [32] up to 18 Å), while the fourth class represents non-contacts and is still the most numerous. Although this definition is somewhat arbitrary, our results were only minimally sensitive to small changes in the thresholds. Distances are measured between C_*α *_atoms, as these are the only ones we model during reconstruction. Table 12 shows the class distribution of both types of map in the dataset (reduced, training version, in brackets). It is clear from this table is that the class distribution is more balanced in the 4 class problem, although the last (non-contact) class is, in both cases, by far the most numerous.

**Table 12:** Data set composition

	class 0	class 1	class 2	class 3
8 Å	4,791,529	95,959,036		
	(2,250,011)	(66,985,320)		
Multi class	4,791,529	10,469,018	18,872,624	66,617,394
	(2,250,011)	(5,453,212)	(10,707,356)	(50,824,752)

Number of pairs of residues contained in 8 Å binary classes and the four classes in the Multi class definition in the S3129 dataset. In brackets, numbers for training (only the 2,452 proteins of 200 residues or shorter are used).

#### 3D reconstruction dataset

The protein data set used in reconstruction simulations consists of a non redundant set of 258 protein structures (S258) showing no homology to the sequences employed to train the contact map predictors. This set includes proteins of moderate size (51 to 200 amino acids) and diverse topology as classified by SCOP (Structural Classification of Proteins database) [49] (all-*α*, all-*β*, *α/β*, *α *+ *β*, surface, coiled-coil and small). No two proteins in this set share more than 25% sequence identity.

#### Template generation

For each of the proteins we search for structural templates in the PDB. Templates are obtained by running a round of PSI-BLAST against the PDB (available on March 25th, 2008) using a PSSM generated against the NR database (see Datasets section) with an expectation cutoff of 10.

An obvious problem arising is that many proteins in the set are expected to be in PDB (barring name changes), and every protein that is in the PDB will have a perfect template. To avoid this, we exclude from the results every protein that appears in S3129 or that shows more than 95% identity to any protein in S3129.

The distribution of sequence identity to the best template, and average template identity is plotted in Figure 7. Roughly 15% of the proteins have no hits at more than 10% sequence identity. About 17% of all proteins have at least one very high quality (better than 90% identity) entry in their template set. Although the distribution is not uniform, all identity intervals are adequately represented: for about 44% of the proteins no hit is above 30%; for nearly 17% of the proteins the best hit is in the 30–50% identity interval. The average identity for all PDB hits for each protein, not surprisingly, is generally low: for roughly 75% of all proteins the average identity is below 30%.

In the case of CASP7 results, we run the same protocol for generating templates, but use the version of the PDB available on April 22nd 2006, roughly two weeks before the beginning of the CASP7 experiment, so we do not make use of any templates that would not have been available at the time.

It should be noted that template generation is an independent module in the systems. We are currently investigating whether more subtle strategies for template recognition would still benefit contact map predictions, with or without retraining the systems on the new template distributions.

### Training/testing protocol

The predictors of contact maps rely on predictions of secondary structure, solvent accessibility and contact density [38]. True structural information was used for training in both ab initio and template based systems. For testing, we used predictions from our servers: Porter [39], PaleAle [33] and BrownAle [38] predicting secondary structure, solvent accessibility and contact density respectively. The ab initio models use ab initio secondary structure, solvent accessibility and contact density predictions. The template models use template-based secondary structure, solvent accessibility and contact density predictions. All our experiments are carried out in 5-fold cross validation. The same dataset and multiple alignments are used to train the ab initio and template based secondary structure predictor Porter, solvent accessibility predictor PaleAle and the contact density predictor BrownAle. By design, these were trained using the same 5 fold split as the map predictors, therefore removing a trained fold while testing was a simple procedure and all 1D predictions are by models that were trained on a dataset independent on the query.

### Measures of prediction quality

To measure the quality of the predictions we use Accuracy, Coverage and their harmonic mean, F1. In particular, Coverage for class *k *(C_*k*_) is the total number of residue pairs correctly assigned to *k*, divided by the total number of residue pairs *observed *in *k*, while Accuracy for class *k *(A_*k*_) is equal to the total number of residue pairs correctly assigned to *k *divided by the total number of residue pairs *predicted *in *k*. F1 for class *k *is:

(9) $$F1_{k}=\frac{2A_{k}C_{k}}{A_{k}+C_{k}}$$

Figures 1, 2, 3 report F1 for the 8 Å contact class (i.e. for pairs closer than 8 Å).

Results in the various tables are instead Q_2 _or Q_4 _(for binary and 4-class, respectively), defined as the total number of correctly predicted residue pairs divided by the total number of residue pairs, no matter what class they are in.

For the CASP7 results, in order to obtain maps with roughly the correct number of contacts, we aim for Accuracy ≈ Coverage at each sequence separation for our method. This is determined by predicting, for each sequence separation, as many contacts as the sum of the estimated probabilities of contact. For the algorithms by other groups we tried three different assignments of contacts: one in which all residue pairs submitted to CASP are considered contacts; one in which we consider only contacts with a reported confidence greater than 0.5; one in which we apply the same rule we used for our predictor. For all methods we choose the one that gives the highest F1 which, in all cases, is the first assignment (i.e. all pairs submitted are to be considered contacts).

## Authors' contributions

IW designed, trained and tested the contact and distance map predictors. DB designed the reconstructor of C_*α *_traces and ran the reconstruction experiments. AJMM helped with the definition of the problem, the design of the set and the CASP7 analysis. CM built the training and testing sets, and the pipeline for homology detection and analysed part of the results. AV and GP contributed design and experimental ideas for all stages. GP developed the basic algorithmic idea for incorporating templates. All authors have contributed to the manuscript and approve its contents.

## References

[B1] Chandonia J, Brenner S (2006). The Impact of Structural Genomics: Expectations and Outcomes. Science.

[B2] Adams M, Joachimiak A, Kim GT, Montelione R, Norvell J (2004). Meeting review: 2003 NIH protein structure initiative workshop in protein production and crystallization for structural and functional genomics. J Struct Funct Genomics.

[B3] Moult J, Fidelis K, Rost B, Hubbard T, Tramontano A (2005). Critical Assessment of Methods of Protein Structure Prediction (CASP) – Round 6. Proteins.

[B4] Bates P, Kelley L, MacCallum R, Sternberg M (2001). Enhancement of protein modeling by human intervention in applying the automatic programs 3D-JIGSAW and 3D-PSSM. Proteins.

[B5] Zhou H, Pandit S, Borreguero J, Chen H, Wroblewska L, Skolnick J (2007). Analysis of TASSER-based CASP7 protein structure prediction results. Proteins.

[B6] Cheng J (2008). A multi-template combination algorithm for protein comparative modeling. BMC Structural Biology.

[B7] CASP Home page. http://predictioncenter.org/.

[B8] Larranaga P, Calvo B, Santana R, Bielza C, Galdiano J, Inza I, Lozano JA (2006). Machine learning in bioinformatics. Briefings in bioinformatics.

[B9] Simons KT, Kooperberg C, Huang E, Baker D (1997). Assembly of protein tertiary structures from fragments with similar local sequences using simulated annealing and Bayesian scoring functions. J Mol Biol.

[B10] Vendruscolo M, Kussell E, Domany E (1997). Recovery of protein structure from contact maps. Folding and Design.

[B11] Fariselli P, Casadio R (1999). A neural network based predictor of residue contacts in proteins. Protein Engineering.

[B12] Fariselli P, Casadio R (2000). Prediction of the number of residue contacts in proteins. Proc Int Conf Intell Syst Mol Biol.

[B13] Fariselli P, Olmea O, Valencia A, Casadio R (2001). Prediction of contact maps with neural networks and correlated mutations. Protein Engineering.

[B14] Pollastri G, Baldi P (2002). Prediction of Contact Maps by Recurrent Neural Network Architectures and Hidden Context Propagation from All Four Cardinal Corners. Bioinformatics.

[B15] Shao Y, Bystroff C (2003). Predicting interresidue contacts using templates and pathways. Proteins.

[B16] Zhao Y, Karypis G (2003). Prediction of contact maps using support vector machines. 3rd international conference on Bioinformatics and Bioengineering (BIBE).

[B17] Pollastri G, Baldi P, Vullo A, Frasconi P (2003). Prediction of Protein Topologies Using GIOHMMs and GRNNs. Advances in Neural Information Processing Systems (NIPS) 15, MIT Press.

[B18] McCallum R (2004). Striped sheets and protein contact prediction. Bioinformatics.

[B19] Punta M, Rost B (2005). PROFcon: novel prediction of long-range contacts. Bioinformatics.

[B20] Cheng J, Baldi P (2007). Improved Residue Contact Prediction Using Support Vector Machines and a Large Feature Set. BMC Bioinfomatics.

[B21] Ortiz A, Kolinski A, Rotkiewicz P, Ilkowski B, Skolnick J (1999). Ab initio folding of proteins using restraints derived from evolutionary information. Proteins.

[B22] Punta M, Rost B (2005). Protein folding rates estimated from contact predictions. Journal of Molecular Biology.

[B23] Schlessinger A, Punta M, Rost B (2007). Natively unstructured regions in proteins identified from contact predictions. Bioinformatics.

[B24] Pazos F, Helmer-Citterich M, Ausiello G, Valencia A (1997). Correlated mutations contain information about protein-protein interaction. Journal of Molecular Biology.

[B25] Izarzugaza JMG, Grana O, Tress ML, Valencia A, Clarke ND (2007). Assessment of intramolecular contact predictions for CASP7. Proteins.

[B26] Wu S, Zhang Y (2008). A comprehensive assessment of sequence-based and template-based methods for protein contact prediction. Bioinformatics.

[B27] Bau D, Pollastri G, Vullo A Analysis of Biological Data: A Soft Computing Approach, World Scientific 2007 chap Distill: a machine learning approach to ab initio protein structure prediction.

[B28] Vassura M, Margara L, Di Lena P, Medri F, Fariselli P, Casadio R (2008). Reconstruction of 3D Structures From Protein Contact Maps. IEEE/ACM Trans Comput Biol Bioinform.

[B29] Zhang Y, Skolnick J (2004). Scoring function for automated assessment of protein structure template quality. Proteins.

[B30] Aszodi A, Gradwell M, Taylor W (1995). Global fold determination from a small number of distance restraints. J Mol Biol.

[B31] Lund O, Frimand K, Gorodkin J, Bohr H, Bohr J, Hansen J, Brunak S (1997). Protein distance contraints predicted by neural networks and probability density functions. Pro Eng.

[B32] Vassura M, Margara L, Di Lena P, Medri F, Fariselli P, Casadio R (2008). FT-COMAR: fault tolerant three-dimensional structure reconstruction from protein contact maps. Bioinformatics.

[B33] Pollastri G, Martin A, Mooney C, Vullo A (2007). Accurate prediction of protein secondary structure and solvent accessibility by consensus combiners of sequence and structure information. BMC Bioinformatics.

[B34] Shackelford G, Karplus K (2007). Contact prediction using mutual information and neural nets. Proteins.

[B35] Baldi P, Pollastri G (2003). The Principled Design of Large-Scale Recursive Neural Network Architectures – DAG-RNNs and the Protein Structure Prediction Problem. Journal of Machine Learning Research.

[B36] Bengio Y, Simard P, Frasconi P (1994). Learning Long-Term Dependencies with Gradient Descent is Difficult. IEEE Transactions on Neural Networks.

[B37] Ceroni A, Frasconi P, Pollastri G (2005). Learning Protein Secondary Structure from Sequential and Relational Data. Neural Networks.

[B38] Vullo A, Walsh I, Pollastri G (2006). A two-stage approach for improved prediction of residue contact maps. BMC Bioinformatics.

[B39] Pollastri G, McLysaght A (2005). Porter: a new, accurate server for protein secondary structure prediction. Bioinformatics.

[B40] Pollastri G, Fariselli P, Casadio R, Baldi P (2002). Prediction of Coordination Number and Relative Solvent Accessibility in Proteins. Proteins.

[B41] Hobohm U, Sander C (1994). Enlarged representative set of protein structures. Protein Sci.

[B42] Kabsch W, Sander C (1983). Dictionary of protein secondary structure: pattern recognition of hydrogen-bonded and geometrical features. Biopolymers.

[B43] Rost B, Sander C (1994). Combining evolutionary information and neural networks to predict protein secondary structure. Proteins.

[B44] Riis SK, Krogh A (1996). Improving prediction of protein secondary structure using structured neural networks and multiple sequence alignments. J Comput Biol.

[B45] Jones DT (1999). Protein secondary structure prediction based on position-specific scoring matrices. J Mol Biol.

[B46] Pollastri G, Przybylski D, Rost B, Baldi P (2002). Improving the prediction of protein secondary structure in three and eight classes using recurrent neural networks and profiles. Proteins.

[B47] Pollastri G, Baldi P (2002). Prediction of Contact Maps by Recurrent Neural Network Architectures and Hidden Context Propagation from All Four Cardinal Corners. Bioinformatics.

[B48] Altschul S, Madden T, Schaffer A (1997). Gapped blast and psi-blast: a new generation of protein database search programs. Nucl Acids Res.

[B49] Andreeva A, Howorth D, Brenner S, TJP H, Chothia C, Murzin A (2004). SCOP database in 2004: refinements integrate structure and sequence family data. Nucl Acid Res.

